# Computer-Based Tools for Diagnosis and Treatment of Alcohol Problems

**Published:** 2006

**Authors:** Reid K. Hester, Joseph H. Miller

**Affiliations:** Reid K. Hester, Ph.D., is director of the Research Division at Behavior Therapy Associates, LLP, Albuquerque, New Mexico. Joseph H. Miller, M.S.W., is a research associate at Behavior Therapy Associates, LLP, and a senior research scientist at the University of New Mexico’s Center on Alcoholism, Substance Abuse, and Addictions, in Albuquerque, New Mexico

**Keywords:** health services research, alcoholism treatment services research, health care costs, social and economic cost of AODU (alcohol and other drug use), economic cost and benefit of AOD, AODU cost estimation problem, cost-effectiveness of AOD health services, treatment programs

## Abstract

Diagnosis and treatment of alcohol-related problems are time-intensive procedures that often are difficult to implement in busy clinical settings. Computer-based tools are one approach that may enhance the availability and cost-effectiveness of assessment and intervention and also may offer other advantages over face-to-face interventions. Several PC- and Internet-based programs have been developed that can be used for assessing alcohol problems, some of which are based on existing screening instruments. Other programs have demonstrated effectiveness as interventions, serving to increase patient motivation and reduce alcohol-associated harm through skill building. Investigators also have begun to analyze the mechanisms through which computer-based programs can induce these effects. Future efforts should be aimed at developing and evaluating additional computer-based interventions, particularly for specific patient subgroups, and at removing barriers to the incorporation of such programs into clinical practice.

Computer-based assessments and interventions are a promising avenue for increasing the accessibility and cost-effectiveness of treatment for alcohol-related problems. This article describes the advantages of computer-based tools and reviews those assessment and intervention programs that have been evaluated in controlled clinical trials, the results of which have been published in peer-reviewed journals. With programs used for assessment and diagnosis of alcohol-related problems, it is particularly important to focus on instruments that have acceptable psychometric properties (i.e., those demonstrated to reliably measure certain psychological variables). Few PC- and Internet-based programs that provide treatment interventions meet this criterion. Moreover, although evidence attests to the effectiveness of some computer-based interventions, their cost-effectiveness has not been formally studied. (For a qualitative description of the costs of some computer-based interventions, see the textbox.)

## Advantages of Computer-Based Tools

PC- or Web-based software programs have several advantages over face-to-face interventions:

- They require little or no therapist involvement, which may increase cost-effectiveness.
- They can provide personalized feedback to clients in a timely and visually engaging manner.
- They can minimize bias that could arise in clinical relationships (i.e., provider–client interactions) (Miller and Sovereign 1989).
- They can more consistently provide specific recommendations for change, based on the results of the assessment and other empirical evidence.
- They offer the ability to store information in a database format so that it can be studied and analyzed by a supervising therapist or health care provider.
- At least some programs allow for automatic collection of followup data and for the generation of outcome reports.
- A given assessment or treatment program can be widely disseminated while maintaining treatment fidelity.

These advantages are supported by research demonstrating that personal data derived from computer-based interviews in the psychiatry and alcohol and other drug (AOD) abuse fields are accurate and valid. Almost 30 years ago, Greist (1977) noted that as the information solicited becomes more sensitive, clients preferred a computer-based interview. And Servan-Schreiber (1986) concluded that “patients suffering from … alcohol or drug abuse tend to report more information to a computer interviewer than to its human counterpart” (p. 197). These findings were corroborated by a report that computer-based screening detected alcohol abuse at twice the rate of face-to-face screening (Kobak et al. 1997). Similarly, Turner and colleagues (1998) demonstrated that in a national survey of teenagers, self-reported rates of high-risk health behaviors were significantly higher when an audio-computer-assisted self-interviewing program was used than when a traditional paper-and-pencil questionnaire was used. Finally, Paperny and Hedberg (1999) documented significantly higher levels of self-reports of high-risk behaviors in teens receiving preventive health services when screening was performed using a computer program.

## Tools for Assessing Alcohol Problems

Several screening and assessment instruments now are available in computer- or Web-based versions. Twenty-eight instruments that offer computerized versions, 21 of which are self-administered, are listed in the second edition of *Assessing Alcohol Problems: A Guide for Clinicians and Researchers*, published by the National Institute on Alcohol Abuse and Alcoholism (NIAAA) (Allen and Wilson 2004). As Allen (2004) has pointed out, these alternative methods of administration may be more engaging for clients and may increase response accuracy.

Two commonly used screening instruments are the Alcohol Use Disorders Identification Test (AUDIT) and the CAGE. These instruments are listed in the NIAAA *Guide* and are available in computerized versions. The AUDIT also is available in Web-based versions (e.g., at www.drinkers-checkup.com and www.alcoholscreening.org).

In addition, the NIAAA *Guide* lists 18 diagnostic tools, many of which allow for computerized scoring (e.g., the Alcohol Dependence Scale [ADS], Composite International Diagnostic Interview [CIDI core] Version 2.1, Diagnostic Interview Schedule for DSM–IV [DIS–IV], or Substance Dependence Severity Scale [SDSS]).

The Addiction Severity Index–Multimedia Version (ASI–MV) is another good example of a commonly used diagnostic instrument that is available as a self-administered computerized version. The ASI–MV is a PC-based program that leads the user through a series of video interviews corresponding to the domains assessed in the ASI. Both the questions and the answer options are read aloud, and the answers are highlighted as they are read. The user responds by pointing the mouse and clicking on the most appropriate answer. With this design, the user does not need to be literate or computer literate. In a study of the ASI–MV’s psychometric properties, users described the program as easy to use and engaging (Butler at al. 2001). In the same study, the program’s developers found good-to-superior psychometric qualities for the ASI–MV (Butler et al. 2001). With the interviewer-administered version of the ASI, investigators had noted that it was difficult to ensure that different interviewers interpreted the results in the same manner (i.e., to maintain a high degree of inter-rater reliability). Because the rating in the computer-based version is done automatically, the ASI–MV is an attractive option for treatment providers who already use the ASI in developing treatment plans. The ASI–MV program requires a Windows-driven PC with a CD–ROM drive and speakers.

## Tools for Interventions

Far less research has been done on computerized interventions for alcohol problems than on computerized screening and diagnosis. Although both PC- and Web-based brief interventions have been developed, very few have undergone formal testing for efficacy. Linke and colleagues (2004) described a pilot Web-based effort targeting a general population in the United Kingdom. In the United States, Web-based intervention efforts have been aimed primarily at college students (for a review, see Walters et al. 2005). For example, Kypri and colleagues (2004) published positive findings about a Web-based application called e-SBI that targets a college audience.

### Increasing Motivation With Brief Interventions

Hester and colleagues (2005) have developed what appears to be the only available computer-based brief motivational intervention with published evidence of efficacy in a population of problem drinkers. This intervention, called the Drinker’s Check-Up (DCU), is a computer-based version of the face-to-face brief motivational intervention protocol by the same name developed by Miller and colleagues (1988). The DCU, which can be used either as a stand-alone intervention or as a prelude to longer-term inpatient or outpatient treatment (see Brown and Miller 1993), consists of three modules:

- Module 1 assesses the user’s drinking, risk factors, alcohol-related problems, symptoms of dependence, and motivation.
- Module 2 provides personalized, objective feedback based on the user’s responses.
- Module 3 helps the user resolve his or her ambivalence about changing drinking patterns.

The computer-based version of the DCU is available both as a Windows program and as an Internet application (www.drinkerscheckup.com). The Windows-based version also offers a followup program (the Follow-up Drinker’s Check-Up [FDCU]) that allows providers to collect data at up to three followup points. The FDCU can generate outcome reports for individual users, user subgroups, and the total sample in a database, making it useful as a program evaluation tool.

The DCU was evaluated as a stand-alone intervention in a randomized clinical trial with a 12-month followup (Hester et al. 2005; Squires and Hester 2004). In the study, 61 problem drinkers recruited from the community were randomly assigned either to a group that received the intervention immediately or to a group that received the intervention after a 4-week waiting period. Overall, participants in both groups reduced the quantity and frequency of drinking by 50 percent after receiving the intervention and showed similar reductions in alcohol-related problems and symptoms of dependence that were sustained through the 12-month followup.

The Windows version of the DCU/FDCU requires a Windows-driven PC and a printer. Primary health care settings that screen patients for alcohol problems may find the DCU/FDCU helpful either as a stand-alone tool or as the first step in a stepped-care model of treatment for their patients. AOD abuse treatment providers could use the DCU program as a prelude to skills-based interventions and the FDCU for collecting followup data and for program evaluation.

**Program Costs**

Although evidence indicates that some computer-based interventions are effective, there has been no formal study of their effectiveness in relation to their cost. To provide researchers and treatment providers with some means of evaluating the cost-effectiveness of these programs, the costs associated with the use of some of these programs are listed here.

- The Addiction Severity Index–Multimedia Version (ASI–MV). This program costs $199 and can be used with five clients. Additional uses cost $5–8 each, depending on the quantity ordered.
- The Drinker’s Check-Up/Follow-Up Drinker’s Check-Up. The purchase price of the DCU/FDCU program is $400, and there are no additional user fees. The Internet version available at www.drinkerscheckup.com costs $25 per user. A Spanish version is available at http://www.drinkerscheckup.com/index_spanish.htm.
- The Behavioral Self-Control Program for Windows (BSCPWIN)–Therapist’s Version. This program costs $500 and has no per-use fees. It comes with a license to provide copies of the Single User version to the therapist’s clients without further charge. The Single User version’s cost to the general public is $25.

It is important to note that the total costs of a given computer-based assessment or intervention tool include not only the program’s purchase price and any per-use fees, but also the time necessary to train counselors in using the program as well as the cost associated with implementing the program and integrating it into the workflow of a clinic. These indirect costs are not discussed in the literature but should be considered in any analysis of cost-effectiveness.

### Harm Reduction Through Skill-Building

The Behavioral Self-Control Program for Windows (BSCPWIN) is a skills-oriented program that helps less severely dependent drinkers learn moderate drinking skills. It consists of eight modules that focus on setting goals, self-monitoring, controlling the drinking rate, setting up rewards and penalties, developing alternatives to drinking, identifying high-risk situations, preventing relapse, and a final progress review and feedback. After users have completed the self-monitoring component, each time users return to the program it asks them to enter their self-monitored alcohol consumption data and gives them feedback on their drinking relative to the goals they set previously. The program then continues with the next module in the sequence.

The BSCPWIN program was evaluated as a stand-alone intervention in a randomized clinical trial with a 12-month followup. Forty heavy drinkers were randomly assigned to receive the BSCPWIN program either immediately or after a 10-week waiting period (Hester and Delaney 1997). Drinking outcomes assessed at 10 weeks, 20 weeks, and 12 months supported the effectiveness of the program. At 10 weeks, the Immediate group had reduced their total drinks per week by 61 percent (from 35.2 to 13.8 drinks per week) and their estimated peak blood alcohol concentrations by 66 percent (from 0.180 percent to 0.060 percent). The Waitlist group did not change their drinking behavior until they received the intervention; after that, they exhibited declines in alcohol consumption at 20 weeks comparable to those the Immediate group had shown at 10 weeks. These outcomes were maintained through the 12-month followup. The accuracy of these self-reports was supported by corroboration from the subjects’ significant others. Moreover, the standardized effect sizes for drinking variables were comparable to those found in studies in which the BSCP protocol was delivered in a face-to-face format. Finally, the study found some evidence of a concurrent decline in other recent drug use among the participants. At baseline, 40 percent of the participants had reported recent use of other drugs. This number declined to 30 percent at the 12-month followup.

BSCPWIN is available both in a Therapist’s version and a Single User version, which can be given to a therapist’s clients or obtained by the general public. Primary health care settings that screen patients for alcohol problems may find the BSCPWIN helpful in a stepped-care model of treatment for their patients. In addition, the program may be useful for AOD abuse treatment providers whose patient population includes less severely dependent drinkers.

## How Do Computer-Based Programs Increase Motivation and Behavioral Changes?

The outcome data from the studies described in the previous section support the effectiveness of computer-based interventions in the treatment of people with alcohol problems. But what makes these interventions effective is another question. How do software programs and Web-based applications motivate people to change and help them learn the skills necessary to change their behavior successfully? As yet, no solid data are available to answer this question conclusively. A review by Walters and Neighbors (2005) of PC- and Web-based brief interventions for college students suggests, however, that personalized feedback may be a key component. Both intervention programs described above provide some personalized feedback, either about people’s current drinking (DCU) or about their progress in changing their drinking (BSCPWIN). The degree to which the information presented is customized or tailored to the individual user also may be key. Schneider and colleagues (1990) found with an intervention for smoking cessation that users were more likely to succeed if they received more customized and tailored messages.

## Conclusions

There is both good news and bad news concerning computer-based tools for assessing and intervening with patients with alcohol problems. The good news is that a growing number of PC- and Web-based screening and diagnostic instruments with good psychometric properties are available. The bad news is that there are only a few programs for conducting interventions with clients. However, randomized clinical trials published in peer-reviewed journals have found evidence for the effectiveness of the few available intervention programs.

Some factors may interfere with the incorporation of these programs into clinical practice. For example, although the purchase prices for these programs are relatively modest, as noted in the textbox, the costs associated with provider training, implementation, and integration of programs into clinical practice are barriers to their adoption. This issue warrants further analysis.

Perhaps the biggest barrier to more widespread dissemination of computer-based interventions is the reluctance of providers and treatment programs to change their mindset about using computer-based tools with their patients, particularly tools that provide direct PC- or Web-based interventions rather than just assessment. Anecdotal data indicate, however, that participants in clinical trials of the DCU and BSCPWIN liked working with these programs. They also clearly benefited clinically from their use. Furthermore, providers’ positive experiences with the growing number of computer-based assessment tools may increase their willingness to explore the use of computer-based approaches for intervention as well.

Currently, clinicians, their clients, and the general public who are looking for treatment options for alcohol-related problems on the Internet have relatively few choices. Therefore, additional programs are needed that provide brief motivational interventions particularly for people who primarily use drugs other than alcohol, for people with comorbid psychiatric disorders, and for heavy-drinking college students. Also needed are programs for people actively pursuing abstinence goals who want to use scientifically supported protocols. Finally, PC- or Web-based programs should be developed for people who are not in treatment for alcohol-related problems but are interested in changing their drinking behavior.

## References

[b1-36-40] Allen JP, Allen JP, Wilson VB (2004). Assessment of alcohol problems: An overview. Assessing Alcohol Problems: A Guide for Clinicians and Researchers.

[b2-36-40] Allen JP, Wilson VB (2004). Assessing Alcohol Problems: A Guide for Clinicians and Researchers.

[b3-36-40] Brown JM, Miller WR (1993). Impact of motivational interviewing on participation and outcome in residential alcoholism treatment. Psychology of Addictive Behaviors.

[b4-36-40] Butler SF, Budman SH, Goldman RJ (2001). Initial validation of a computer-administered Addiction Severity Index: The ASI–MV. Psychology of Addictive Behaviors.

[b5-36-40] Greist J (1977). Computer measures of patient progress in psychotherapy. Psychiatry Digest.

[b6-36-40] Hester RK, Delaney HD (1997). Behavioral Self-Control Program for Windows: Results of a controlled clinical trial. Journal of Consulting and Clinical Psychology.

[b7-36-40] Hester RK, Squires DD, Delaney HD (2005). The Drinker’s Check-Up: 12-month outcomes of a controlled clinical trial of a stand-alone software program for problem drinkers. Journal of Substance Abuse Treatment.

[b8-36-40] Kobak KA, Taylor LH, Dottl SL (1997). A computer-administered telephone interview to identify mental disorders. JAMA: Journal of the American Medical Association.

[b9-36-40] Kypri K, Saunders JB, Williams SM (2004). Web-based screening and brief intervention for hazardous drinking: A double-blind randomized controlled trial. Addiction.

[b10-36-40] Linke S, Brown A, Wallace P (2004). Down your drink: A Web-based intervention for people with excessive alcohol consumption. Alcohol and Alcoholism.

[b11-36-40] Miller WR, Sovereign RG, Loberg T, Miller WR, Nathan PE, Marlatt GA (1989). The check-up: A model for early intervention in addictive behaviors. Addictive Behaviors: Prevention and Early Intervention.

[b12-36-40] Miller WR, Sovereign RG, Krege B (1988). Motivational interviewing with problem drinkers: II. The Drinker’s Check-Up as a preventive intervention. Behavioural Psychotherapy.

[b13-36-40] Paperny DMN, Hedberg VA (1999). Computer-assisted health counselor visits. Archives of Pediatrics & Adolescent Medicine.

[b14-36-40] Schneider SJ, Walter R, O’Donnell R (1990). Computerized communication as a medium for behavioral smoking cessation treatment: Controlled evaluation. Computers in Human Behavior.

[b15-36-40] Servan-Schreiber D (1986). Artificial intelligence and psychiatry. Journal of Nervous and Mental Disease.

[b16-36-40] Squires DD, Hester RK (2004). Using technical innovations in clinical practice: The Drinker’s Check-Up software program. Journal of Clinical Psychology.

[b17-36-40] Turner CF, Ku L, Rogers SM (1998). Adolescent sexual behavior, drug use, and violence: Increased reporting with computer survey technology. Science.

[b18-36-40] Walters S, Hester RK, Chiauzzi EM, Miller E (2005). Demon rum: High-tech solutions to an age-old problem. Alcoholism: Clinical and Experimental Research.

